# Consequences of a 2-Deoxyglucose Exposure on the ATP Content and the Cytosolic Glucose Metabolism of Cultured Primary Rat Astrocytes

**DOI:** 10.1007/s11064-024-04192-y

**Published:** 2024-06-19

**Authors:** Antonia Regina Harders, Patrick Watermann, Gabriele Karger, Sadhbh Cynth Denieffe, Alina Weller, Annika Carina Dannemann, Johanna Elisabeth Willker, Yvonne Köhler, Christian Arend, Ralf Dringen

**Affiliations:** 1 Centre for Biomolecular Interactions Bremen, Faculty 2 (Biology/Chemistry), University of Bremen, P.O. Box 330440, 28334 Bremen, Germany; 2 Centre for Environmental Research and Sustainable Technologies, University of Bremen, Bremen, Germany

**Keywords:** Astrocytes, ATP, Deoxyglucose, Glucose, Glycolysis, Pentose phosphate pathway

## Abstract

The glucose analogue 2-deoxyglucose (2DG) has frequently been used as a tool to study cellular glucose uptake and to inhibit glycolysis. Exposure of primary cultured astrocytes to 2DG caused a time- and concentration-dependent cellular accumulation of 2-deoxyglucose-6-phosphate (2DG6P) that was accompanied by a rapid initial decline in cellular ATP content. Inhibitors of mitochondrial respiration as well as inhibitors of mitochondrial uptake of pyruvate and activated fatty acids accelerated the ATP loss, demonstrating that mitochondrial ATP regeneration contributes to the partial maintenance of the ATP content in 2DG-treated astrocytes. After a 30 min exposure to 10 mM 2DG the specific content of cellular 2DG6P had accumulated to around 150 nmol/mg, while cellular ATP was lowered by 50% to around 16 nmol/mg. Following such a 2DG6P-loading of astrocytes, glycolytic lactate production from applied glucose was severely impaired during the initial 60 min of incubation, but was reestablished during longer incubation concomitant with a loss in cellular 2DG6P content. In contrast to glycolysis, the glucose-dependent NADPH regeneration via the pentose phosphate pathway (PPP) was only weakly affected in 2DG6P-loaded astrocytes and in cells that were coincubated with glucose in the presence of an excess of 2DG. Additionally, in the presence of 2DG PPP-dependent WST1 reduction was found to have doubled compared to hexose-free control incubations, indicating that cellular 2DG6P can serve as substrate for NADPH regeneration by the astrocytic PPP. The data presented provide new insights on the metabolic consequences of a 2DG exposure on the energy and glucose metabolism of astrocytes and demonstrate the reversibility of the inhibitory potential of a 2DG-treatment on the glucose metabolism of cultured astrocytes.

## Introduction

2-Deoxyglucose (2DG) is an artificial glucose analog that is taken up into cells via glucose transporters (GLUTs) and is phosphorylated in many cells by hexokinase to 2-deoxyglucose-6-phosphate (2DG6P) [1, 2]. Due to the missing hydroxyl group at carbon 2, 2DG6P cannot be isomerized by hexose phosphate isomerase to the respective ketose and accumulates in cells. The rapid accumulation of 2DG6P has frequently been used in the last century for measuring the local cerebral glucose utilization in vivo after application of ^14^C-2DG [3]. 2DG6P is the predominant metabolite of 2DG in the brain, but also other 2DG metabolites were found in brain lysates including 6-phospho-2-deoxygluconate [4], suggesting that oxidation of 2DG6P by glucose-6-phosphate dehydrogenase (G6PDH) takes place in the brain. In addition, 2DG-1-phosphate was found in brains of 2DG-treated rats [4], consistent with the reported incorporation of 2DG into glycogen [5].

In brain, astrocytes are essential partners of neurons and have a broad range of important functions including the maintenance of the extracellular homeostasis as well as in providing metabolic support and defense against oxidative stress and xenobiotics [6–9]. As astrocytes cover the brain capillaries almost completely with their end-feet [10], astrocytes are the first parenchymal cell type of the brain that will be exposed to 2DG that is transported from the periphery into the brain via the blood.

Several reports have previously described various aspects of 2DG metabolism for cultured astrocytes. These cells are able to take up 2DG [11, 12] which is rapidly phosphorylated to 2DG6P [13, 14]. Whether astrocytes release 2DG from the accumulated cellular 2DG6P is a matter of debate as contradictory data have been reported on this question [13, 14]. During exposure of astrocytes with 2DG glycogen mobilization is prevented due to the inhibition of glycogen phosphorylase by 2DG6P [15]. A 2DG-treatment of cultured astrocytes for 24 h does not appear to compromise cell viability of astrocytes [16–18], but contaminating microglial cells in the cultures did not survive this treatment [16], consistent with the reported lowered inflammatory signaling of such cultures [17].

Exposure of astrocytes to 2DG has consequences on the cellular ATP content as demonstrated by the reduction in cellular ATP content by 40% within 20 min [19]. However, even after 24 h incubation of glucose-deprived astrocytes with 2DG, around 30% of the initial cellular ATP content was detectable and the cells remained viable [18]. As astrocytes regenerate ATP by both glycolysis and mitochondrial oxidative phosphorylation [18], a co-incubation of the glycolysis inhibitor 2DG with inhibitors of the respiratory chain strongly accelerates the loss in cellular ATP as reported for the co-applications of 2DG with the complex I inhibitor rotenone [19] or with the complex III inhibitor antimycin A [20].

Due to its potential as glycolysis inhibitor, 2DG is considered for treatments of human patients as anticancer drug [1, 21]. In addition, due to its acute anti-seizure effects in several model systems, application of 2DG has been suggested as potential treatment for status epilepticus [22, 23]. In order to better understand the consequences of a 2DG treatment on the metabolism of brain cells, we exposed cultured primary rat astrocytes to 2DG and investigated the effects of such a treatment on the cellular ATP content as well as on the functions of glycolysis and pentose phosphate pathway (PPP). Here we report that 2DG causes a time- and concentration-dependent partial loss in cellular ATP which was accelerated by inhibition of mitochondrial metabolism or uptake of mitochondrial substrates. 2DG exposure caused a strong cellular accumulation of 2DG6P that impaired glycolytic lactate production from glucose, while the NADPH regeneration via the PPP that is needed for WST1 reduction was less severely affected by the accumulated 2DG6P. These effects of a 2DG exposure of astrocytes were transient, consistent with a disappearance of most of the accumulated 2DG6P during further incubations in the absence of 2DG. Finally, some NADPH regeneration that was supporting WST1 reduction was observed in the presence of 2DG, suggesting that accumulated 2DG6P can serve as substrate of G6PDH in astrocytes.

## Materials and Methods

### Materials

2-Deoxy-d-glucose (2DG), antimycin A, BAM15, fetal calf serum (FCS), oligomycin and rotenone were purchased from Sigma-Aldrich (Steinheim, Germany; RRID:SCR_008988). Bovine serum albumin, dimethyl sulfoxide (DMSO), perchloric acid, NAD^+^, NADH and NADP^+^ were from AppliChem (Darmstadt, Germany; RRID:SCR_005814). β-lapachone was from Abcam (Berlin, Germany; RRID:SCR_012931) and WST1 from Dojindo (Munich, Germany). Etomoxir and UK5099 were obtained from Merck (Darmstadt, Germany; RRID:SCR_001287). Dulbecco’s modified Eagles medium (DMEM) and penicillin G/streptomycin sulfate solution were obtained from Thermo Fisher Scientific (Schwerte, Germany; RRID:SCR_008452). The Cell Titer Glo® 2.0 ATP Assay Kit was obtained from Promega (Walldorf, Germany; RRID:SCR_006724). ATP and the enzymes used for assays to quantify lactate, glucose, 2DG and 2DG6P were purchased from Roche Diagnostics (Mannheim, Germany; RRID:SCR_001326). All other basal chemicals were obtained from Carl Roth (Karlsruhe, Germany; RRID:SCR_005711), Sigma-Aldrich (Steinheim, Germany; RRID:SCR_008988), Riedel-de Haën (Seelze, Germany) or Fluka (Buchs, Switzerland). Sterile cell culture materials as well as transparent and black microtiter plates were obtained from Sarstedt (Nümbrecht, Germany).

### Primary Astrocyte Cultures

Astrocyte-rich primary cultures were prepared from the entire brains of newborn Wistar rats as previously described in detail [24]. The rats had been purchased from Charles River Laboratories (Sulzfeld, Germany; RRID:SCR_003792) and were treated in accordance to the State of Bremen, German and European animal welfare acts. The harvested cells were seeded in a density of 300,000 viable cells per well of 24-well dishes in 1 mL culture medium (90% DMEM containing 25 mM glucose, 44.6 mM sodium bicarbonate, 1 mM pyruvate, 20 U/mL penicillin G, 20 µg/mL streptomycin sulfate, supplemented with 10% FCS) and cultured in a Sanyo (Osaka, Japan) CO_2_ incubator in a humidified atmosphere containing 10% CO_2_. The culture medium was renewed every seventh day and one day prior to experiments. The cultures used for the current study had an age between 14 and 28 days. Astrocyte-rich primary cultures are strongly enriched in glial fibrillary acidic protein-positive astrocytes and contain only low numbers of other types of brain cells, including microglial cells, oligodendrocytes, and ependymal cells [24, 25].

### Experimental Incubation of the Cells

All experiments were performed on confluent primary astrocyte cultures in wells of 24-well dishes. The cells were washed twice with 1 mL pre-warmed (37 °C) glucose-free incubation buffer (IB; 145 mM NaCl, 20 mM HEPES, 5.4 mM KCl, 1.8 mM CaCl_2_, 1 mM MgCl_2_, 0.8 mM Na_2_HPO_4_, pH adjusted with NaOH to 7.4 at 37 °C) and subsequently incubated with IB in the absence or the presence of the given concentrations of 2DG and/or other substrates and inhibitors. All incubations were carried out for the time periods indicated in the legends of the figures or the tables at 37 °C in the humidified atmosphere of a CO_2_-free incubator (Sanyo, Japan).

If not stated otherwise, the cells were incubated in 250 µL IB with the given substrates and/or inhibitors indicated for the time periods given in the legends of the figures and tables. For experiments that tested for the consequences of a coincubation of cells with 2DG and glucose, the washed cells were incubated in 300 µL (to test for glycolytic lactate generation) or 400 µL (to test for WST1 reduction) IB containing the given concentrations of glucose plus 10 mM 2DG. For experiments that tested for the consequences of a preloading of cells with 2DG6P, the washed cells were pre-incubated for 30 min in 300 µL (to test for glycolytic lactate generation) or 400 µL (to test for WST1 reduction) glucose-free IB in the absence or the presence of 10 mM 2DG, washed twice with the respective volume of glucose-free IB before the main incubation was performed for the indicated time periods in either 300 µL (to test for glycolytic lactate generation) or 400 µL (to test for WST1 reduction) glucose-free IB that had been supplemented with the substrates and/or inhibitors indicated in the legends of the figures and tables. At the indicated time points the media were collected and 10 µL samples were analyzed for its lactate content and the LDH activity or 50 µL samples for its content of WST1 formazan. The cells were washed twice with 1 mL ice-cold (4 °C) phosphate-buffered saline (PBS; 10 mM potassium phosphate buffer pH 7.4 containing 150 mM NaCl) and lysed for the quantification of cellular contents of ATP, 2DG and 2DG6P.

### Determination of WST1 Reduction

The WST1 formazan content in the incubation media was determined as previously described in detail [26]. The 50 µL samples collected for the given time periods were diluted with water to a total volume of 200 µL in wells of a microtiter plate and the WST1 formazan absorbance was measured at 450 nm in a microtiter plate reader (Multiscan Sky, Thermo Fisher, Darmstadt, Germany) as previously reported [26, 27]. The extracellular WST1 formazan concentration was normalized to the initial protein content of the respective cultures to obtain the specific WST1 formazan content.

### Determination of Extracellular Lactate

The concentration of extracellular lactate after a given incubation period was determined by a coupled enzymatic assay in microtiter plate format as previously described in detail [24]. The lactate present is oxidized by LDH to pyruvate that is subsequently transaminated by glutamate-pyruvate transaminase in an alkaline glutamate buffer. The NADH generated by LDH was determined at 340 nm and allowed the calculation of the concentration of lactate released by the cells [24].

### Determination of Cellular ATP Contents

Perchloric acid lysates of cultured astrocytes were used to quantify cellular ATP contents by a luciferin-luciferase-based luminometric assay as recently described [18, 28]. Briefly, 50 µL of diluted supernatant of KOH-neutralized HClO_4_ lysates or ATP standards (0–1000 nM) were mixed in wells of a black 96-well plate with 50 µL of the ATP detection reagent (Cell Titer Glo® 2.0 ATP Assay Kit). After 20 min of incubation, the luminescence signal was recorded by a microtiter plate reader (Tecan Infinite F200 pro, Tecan, Männedorf, Switzerland). The ATP content of the diluted cell samples was calculated by making use of the linear calibration curve generated from the values determined for the ATP standards. Specific ATP contents were calculated by normalizing the ATP values to the initial cellular protein content of the respective culture.

### Determination of 2DG and 2DG6P

Cellular amounts of 2DG6P and 2DG were determined in neutralized perchloric acid lysates by a modification of a published method [29]. 2DG6P is oxidized to 6-phospho-2-deoxygluconate by G6PDH and the NADPH generated is quantified by the fluorescence signals given for reactions with appropriate standards of 2DG. Briefly, 120 µL of neutralized perchloric acid lysates [28] or 2DG standards (0–40 nmol) in neutralized perchloric acid were mixed in wells of a black microtiter plate with 60 µL of 0.3 M triethanolamine/KOH buffer pH 7.6 and subsequently with 180 µL reaction solution containing 4 mM ATP, 4 mM MgCl_2_, 3 mM NADP^+^, 1.32 U/well G6PDH without (for 2DG6P quantification) or with 0.3 U/well hexokinase (for quantification of 2DG plus 2DG6P) in 0.3 M triethanolamine/KOH buffer pH 7.6. The plate was covered with parafilm and incubated in the dark for 20 h before the fluorescence of the NADPH generated was quantified in the Fluoroskan Ascent FL microtiter plate reader (Thermo Fisher Scientific, Bremen, Germany) by using the 340 nm excitation filter and the 460 nm emission filter. The cellular contents of 2DG plus 2DG6P (reactions with hexokinase) and the content of 2DG6P (reaction without hexokinase) in the lysates were calculated by making use of the linear calibration curve obtained for the 2DG standards (reaction with hexokinase). 2DG contents were calculated by subtracting the values obtained for reactions without hexokinase from those determined in the presence of hexokinase. Specific 2DG6P and 2DG contents were calculated by normalizing the determined values to the initial cellular protein content of the respective culture.

### Determination of Cell Viability and Protein Content

Release of cellular cytosolic LDH was used as an indicator to test for potential cell toxicity of a given treatment. Extracellular LDH activities in media samples were compared with the initial cellular LDH activity to determine a percental toxicity as described previously in detail [24]. The initial cellular LDH activity was determined after lysis of the cells in an appropriate volume (depending on the experiment performed 250 µL, 300 µL or 400 µL) of IB containing 1% (w/v) Triton X-100 (100% LDH release). Ten or 20 µL of incubation media collected after a given treatment or Triton lysates were used as samples to determine the percental LDH release for a given treatment [24]. The initial cellular protein content per well was determined by the Lowry method [30] using bovine serum albumin as standard protein.

### Presentation of Data and Statistical Analysis

The data presented in the figures and tables are means ± standard deviation (SD) of values that were obtained in experiments performed in duplicates on at least three independently prepared astrocyte cultures. Analysis for statistical significance between groups of data was performed by ANOVA followed by the Bonferroni post-hoc test or by the paired t-test. The calculated levels of significance compared to the indicated control conditions are given by *^, +^p < 0.05, **^, ++^p < 0.01 and ***^, +++^p < 0.001. p > 0.05 was considered as not significant.

## Results

### Depletion of Cellular ATP Contents in Cultured Astrocytes After Application of 2DG and/or Inhibitors of Mitochondrial Respiration

**Fig. 1:**
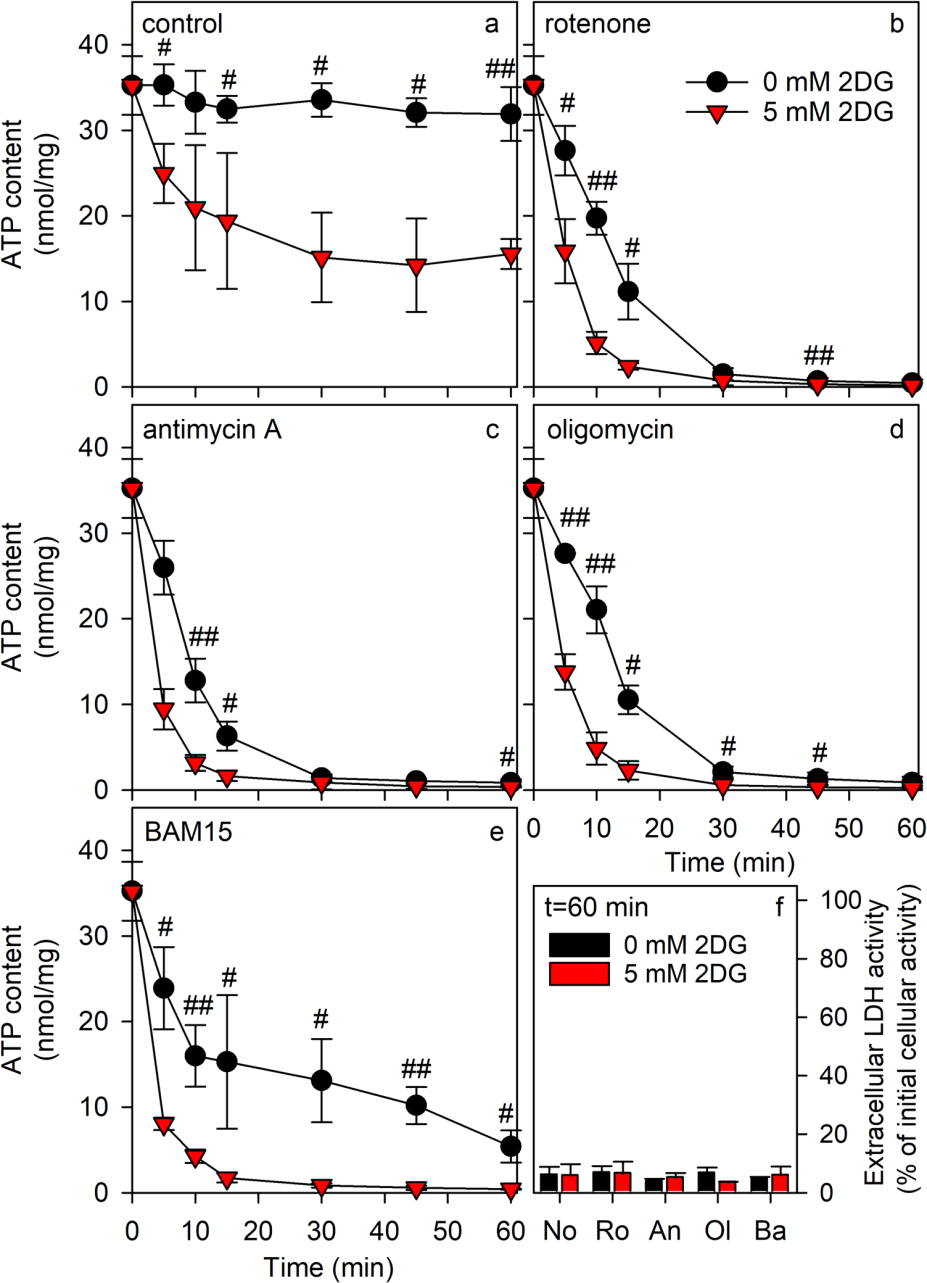
Modulation of ATP contents by 2DG application and/or inhibition of mitochondrial ATP production in primary astrocyte cultures. The cells were incubated in 250 µL IB without (0 mM) or with 5 mM 2DG in the absence (**a**) or the presence of 1 µM rotenone (**b**), antimycin A (**c**), oligomycin (**d**) or BAM15 (**e**) for up to 60 min before the cellular contents of ATP were determined. As indicator for potential loss in membrane integrity the extracellular LDH activity was determined after 60 min (**f**). The presented data are means ± SD of values obtained in three experiments performed on independently prepared cultures. The average initial protein content of the cultures was 117 ± 1 μg/well. The significance of differences (t-test) between data for incubations without and with 2DG are indicated by hashes with ^#^p < 0.05 and ^##^p < 0.01. Abbreviations in panel (**f**): *No* control, *Ro* rotenone, *An* antimycin A, *Ol* oligomycin, *Ba* BAM15

Glycolysis and mitochondrial respiration contribute to the ATP regeneration in cultured astrocytes [18, 20, 31]. To investigate the consequences of an application of the glycolysis inhibitor 2DG and of inhibitors of mitochondrial oxidative phosphorylation on the cellular ATP content, astrocyte cultures were incubated for up to 60 min in glucose-free IB without (0 mM) or with 5 mM 2DG in the absence or the presence of mitochondrial inhibitors. During incubations of astrocytes in the absence of 2DG, the specific ATP content remained unaltered compared to the cellular content at the onset of the incubation (Fig. 1a). In contrast, exposure of astrocytes to 5 mM 2DG caused a rapid decline in cellular ATP level reaching around 50% of the initial ATP content after 30 min (Fig. 1a). During longer incubations of up to 60 min no further decline in ATP contents was observed (Fig. 1a).

Compared to the glycolysis inhibitor 2DG, the applied inhibitors of mitochondrial oxidative phosphorylation depleted the cellular ATP content in glucose-free IB more rapidly and after 30 min of exposure ATP was hardly detectable in cells that had been treated with the complex I inhibitor rotenone [32, 33] (Fig. 1b), the complex III inhibitor antimycin A [20, 34] (Fig. 1c) or the ATP synthase inhibitor oligomycin [35, 36] (Fig. 1d). For cells that had been exposed to the mitochondrial uncoupler BAM15 [28, 37], the cellular ATP content was lowered within 30 and 60 min of incubation to 37% and 15%, respectively, of the initial cellular ATP content (Fig. 1e). Co-application of 2DG with each of the four modulators of mitochondrial metabolism strongly accelerated the decline in cellular ATP content and deprived the cells within 15 min almost completely of their ATP (Fig. 1b–e). None of the applied combinations of 2DG and mitochondrial modulators compromised the cell viability during the 60 min incubation as demonstrated by the absence of any significant increase in extracellular LDH activity (Fig. 1f). In conclusion, the presence of 2DG strongly accelerated the already rapid depletion by mitochondrial inhibitors of the ATP content of glucose-deprived astrocytes.

### Cellular ATP Contents After Exposure of Astrocytes to 2DG and Inhibitors of Mitochondrial Transporters

Oxidation of endogenous mitochondrial substrates such as fatty acids and pyruvate has been demonstrated to contribute to the maintenance of cellular ATP levels in glucose-deprived astrocytes [18]. To test whether such processes may also be involved in the partial maintenance of ATP levels in 2DG-treated astrocytes, the cells were exposed to 5 mM 2DG in the absence or the presence of UK5099, an inhibitor of mitochondrial pyruvate transporter [28, 38] and/or etomoxir, an inhibitor of the uptake of activated long fatty acids into mitochondria [39, 40]. None of the inhibitor treatments caused any obvious toxicity during incubations for up to 180 min as demonstrated by the absence of any increase in extracellular LDH activity (Table 1). In the presence of the inhibitors, no significant further decline in the cellular ATP content was observed during the first 60 min of incubation compared to astrocytes exposed to 2DG only (data not shown). In contrast, for 180 min incubations the co-application of both mitochondrial transport inhibitors, but not the application of one of the two inhibitors alone, significantly lowered the cellular ATP content further to around 20% of the initial ATP content compared to around 40% of the ATP content that was maintained in 2DG-treated control cells (Table 1). Thus, mitochondrial metabolic pathways support the partial maintenance of ATP levels in 2DG-treated astrocytes.

**Table 1:** Effects of mitochondrial uptake inhibitors on the ATP content of 2DG-treated cultured astrocytes

Substrate	ATP content (nmol/ mg)	ATP content (% of initial cellular ATP content)	LDH release (% of initial cellular LDH activity)
None	13.5 ± 1.2	39.7 ± 3.5	7 ± 8
Etomoxir	11.9 ± 1.4	34.9 ± 4.1	12 ± 10
UK5099	13.9 ± 0.5	40.9 ± 1.6	7 ± 10
Etomoxir + UK5099	6.7 ± 3.1**	19.7 ± 9.0**	7 ± 10

The cells were incubated for 180 min in glucose-free IB with 5 mM 2DG in the absence (none) or the presence of etomoxir (30 µM) and/or UK5099 (1 µM) before the cellular ATP content and the extracellular LDH activity were determined. The initial cellular ATP (100% values) and protein contents of the cultures were 34.1 ± 3.6 nmol/mg and 122 ± 15 µg/well, respectively. The specific cellular ATP content of astrocytes that had been incubated in the absence of 2DG accounted after 180 min to 31.5 ± 1.2 nmol/mg. The data shown represent means ± SD of values obtained in experiments performed on three independently prepared cultures

The significance of differences (ANOVA) compared to the control condition (none) is indicated by the symbol **p < 0.01

### ATP Depletion and 2DG6P Accumulation in 2DG-Treated Astrocytes

**Fig. 2:**
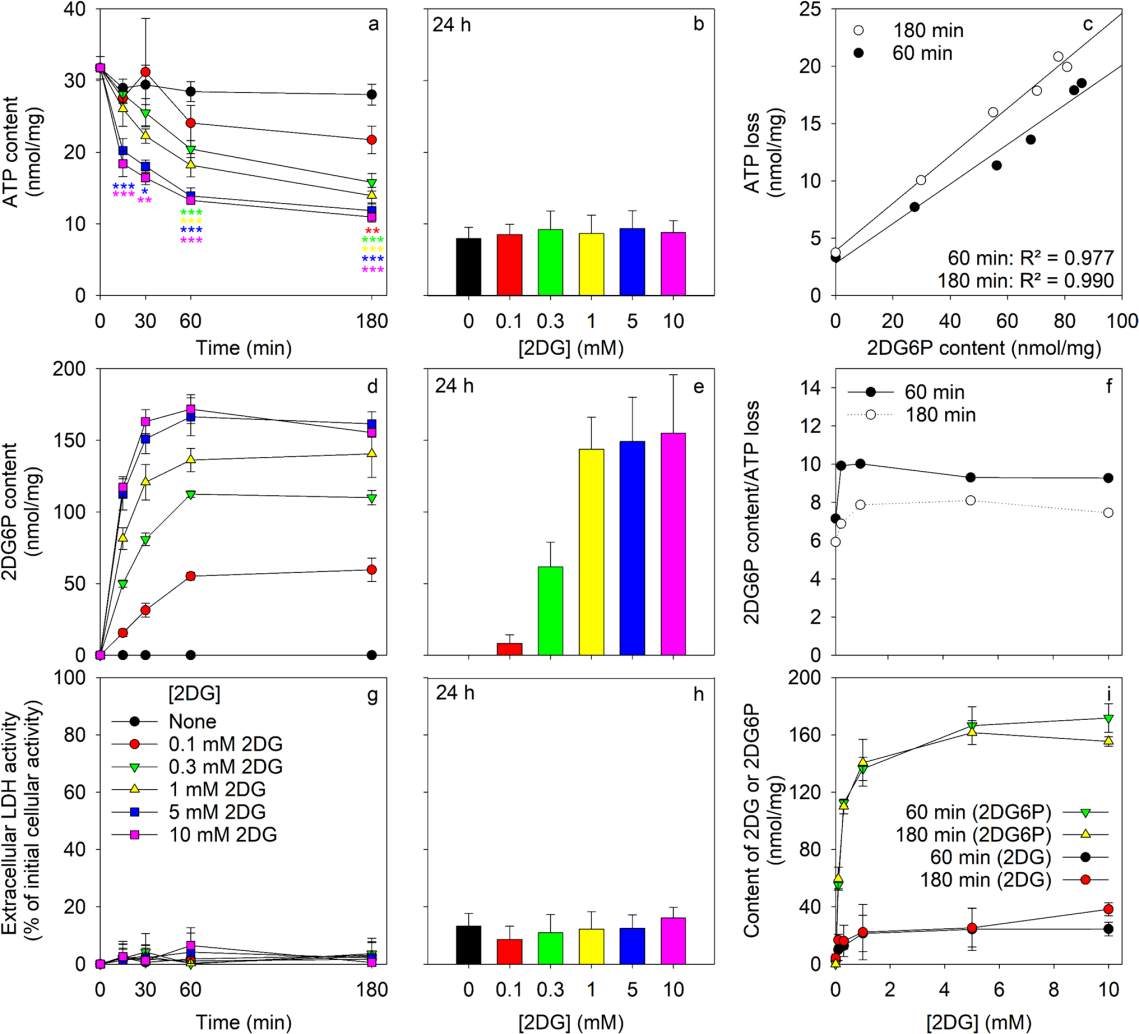
ATP depletion and 2DG6P accumulation in 2DG-exposed cultured primary astrocytes. The cells were incubated for the indicated time periods in 250 µL glucose-free IB in the absence (0 mM) or the presence of the given concentrations of 2DG before the cellular ATP content (**a**, **b**), the cellular 2DG6P content (**d**, **e**) and the extracellular LDH activity (**g**, **h**) were determined. Panel (**c**) shows the correlation of the cellular 2DG6P content to the calculated ATP loss for the 60 min and 180 min incubation period and panel (**f**) the ratio of 2DG6P accumulation to ATP loss for the given 2DG concentrations applied. Panel (**i**) shows the specific cellular contents of 2DG6P and of free 2DG as function of the 2DG concentration applied. The cultures had an initial protein content of 135 ± 19 µg/well. The data are presented as mean values ± SD of values obtained from experiments that were performed on three independently prepared cultures. In panel (**a**), the significance of differences compared to the control condition (absence of 2DG) as calculated by ANOVA are indicated by *p < 0.05, **p < 0.01 and ***p < 0.001

The ATP depletion in 2DG-exposed astrocytes depended strongly on the incubation time and on the concentration of the 2DG applied (Fig. 2a, b). In the absence of 2DG, a high cellular content of ATP was maintained for at least 180 min (Fig. 2a), while the cellular ATP content of glucose-deprived cells declined within 24 h to around 25% of the initial ATP content (Fig. 2b). Already the application of 2DG in the low concentration of 0.1 mM lowered the cellular ATP content significantly by around 30% within 180 min (Fig. 2a). With increasing concentration of 2DG, the initial decline in cellular ATP content was found strongly accelerated for concentration of up to 5 mM 2DG, while 10 mM 2DG did not further accelerate the cellular ATP loss compared to the 5 mM 2DG condition (Fig. 2a). Even for incubations with high 2DG concentrations of 5 mM and 10 mM, cellular ATP contents of around 40% and 35% of the initial cellular ATP content were found maintained after 60 and 180 min, respectively (Fig. 2a). However, after 24 h of incubation, the cellular ATP content was found lowered for all 2DG concentrations applied to values that accounted for around 25% of the initial cellular ATP content and did not differ to that of cells that had been incubated in the absence of 2DG (Fig. 2b). None of the conditions applied caused obvious toxicity as demonstrated by the absence of any substantial increase in extracellular LDH activity (Fig. 2g, h).

After application of 2DG to astrocytes, a strong cellular accumulation of 2DG6P was observed that was dependent on the incubation time and on the 2DG concentration applied (Fig. 2d, e, i). The fast initial 2DG6P accumulation during the first 30 min was slowed during longer incubations. Maximal cellular 2DG6P contents were observed after around 60 min and these contents were not further increased during longer incubations for 180 min (Fig. 2d) or 24 h (Fig. 2e). After 3 h and 24 h of incubation almost identical cellular 2DG6P contents were found for incubations with 2DG in concentrations of 1 to 10 mM, while the low cellular 2DG6P contents determined after 180 min incubation of astrocytes with 0.1 or 0.3 mM 2DG had further declined during an extended incubation for 24 h (Fig. 2d, e).

Correlation of the applied 2DG concentration to the determined steady state contents of 2DG6P revealed a hyperbolic relationship (Fig. 2i) with a half-maximal accumulation of 2DG6P observed for a 2DG concentration of 0.2 mM and a maximal specific accumulation of 170 nmol/mg. In addition to 2DG6P, low amounts of free 2DG were determined in cell lysates of 2DG-treated astrocytes (Fig. 2i) which accounted after 60 min of incubation to values between 10 and 20% of those determined for the respective cellular 2DG6P contents.

After 60 min of incubation of cultured astrocytes with 2DG, both the cellular ATP and 2DG6P contents had reached steady state levels that depended on the initial 2DG concentration applied and were maintained at least for up to 180 min (Fig. 2a, d). Correlation of the amount of ATP that had been lost from the cells during a given 2DG incubation to the cellular content of 2DG6P determined for the respective condition revealed a linear proportionality to the concentration of 2DG applied (Fig. 2c). The ratios of cellular 2DG6P content to the calculated ATP loss for cells that had been exposed to 2DG in concentrations between 0.3 and 10 mM were calculated to be around 10 and 7 for incubation periods of 60 and 180 min, respectively (Fig. 2f). In conclusion, the rapid ATP loss in 2DG-treated astrocytes was accompanied by a much stronger cellular accumulation of 2DG6P.

### Loading of Astrocytes with 2DG6P

To test for the consequences of a high cellular concentration of 2DG6P on metabolic pathways, cultured astrocytes were loaded by a preincubation with 10 mM 2DG for 30 min in different incubation volumes (250 µL, 300 µL or 400 µL) that were used for the different experimental paradigms studied. Cellular 2DG6P had accumulated in the 2DG-treated astrocytes in 30 min to specific contents of 166.2 ± 9.9 nmol/mg (250 µL volume), 151. 9 ± 15.6 nmol/mg (300 µL volume) and 130.6 ± 9.1 nmol/mg (400 µL volume) (Table 2). By using the known specific cytosolic volume of cultured astrocytes of 4.1 µL/mg [41], the cytosolic concentrations of accumulated 2DG6P in 2DG-treated astrocytes was calculated to be around 40 mM (250 µL incubation volume), 37 mM (300 µL incubation volume) and 32 mM (400 µL incubation volume). In addition to 2DG6P, some free 2DG was determined for the lysates (Table 2). For all incubation volumes investigated, the cellular ATP content had been lowered by around 50%, the cell viability had not been compromised (Table 2) and the amount of cellular 2DG6P was below 1% of the initial amount of 2DG applied. In conclusion, after a 30 min incubation with 10 mM 2DG, cultured astrocytes had accumulated 2DG6P in specific contents that corresponded to cytosolic concentrations between 30 and 40 mM. This type of loading with 2DG6P was used in further experiments to test for potential consequences of a high cellular 2DG6P concentration on glycolytic lactate production and PPP activity.

**Table 2:** Cellular contents of ATP, 2DG and 2DG6P after 30 min exposure of cultured astrocytes to 10 mM 2DG in different incubation volumes

Parameter determined	Incubation time (min)	Incubation volume (µL)		
		250	300	400
Number of experiments (n)		4	3	3
Initial protein content (µg/well)	0	125 ± 26	108 ± 11	117 ± 14
Initial ATP content (nmol/mg)	0	32.5 ± 1.5	36.3 ± 4.2	29.7 ± 5.4
ATP content (nmol/mg)	30	16.6 ± 0.8	18.8 ± 1.4	14.6 ± 2.9
2DG6P content (nmol/mg)	30	166.2 ± 9.9	151.4 ± 14.9	130.6 ± 9.1
2DG content (nmol/mg)	30	15.4 ± 21.8	53.1 ± 17.3	26.6 ± 8.1
Extracellular LDH activity (%)	30	3 ± 5	4 ± 6	9 ± 10

The cells were incubated for 30 min with 10 mM 2DG in glucose-free IB before the specific cellular contents of ATP, 2DG6P and free 2DG as well as the extracellular LDH activity (given as % of the initial cellular LDH activity) were determined. The data presented are means ± SD of values from n experiments that had been performed on independently prepared astrocyte cultures

### Glycolytic Lactate Formation and Cellular Contents of ATP and 2DG6P in 2DG-Pretreated Astrocytes

**Fig. 3:**
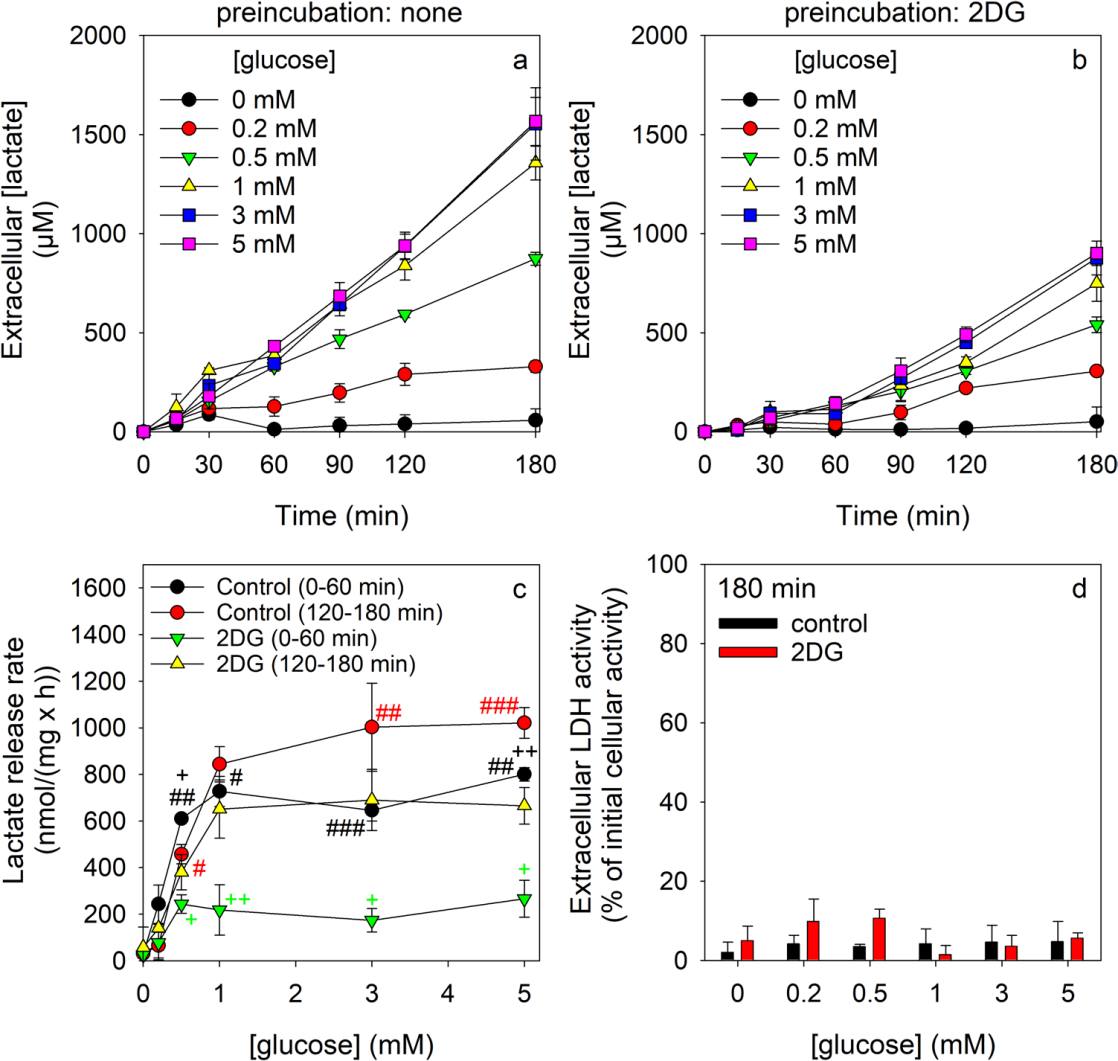
Effects of a 2DG pre-exposure on the lactate release from glucose-fed astrocytes. The cells were pre-incubated for 30 min in 300 µL glucose-free IB without (**a**, **c**, **d**) or with 10 mM 2DG (**b**, **c**, **d**), washed and subsequently incubated with glucose in the indicated concentrations. The extracellular lactate concentration (**a**, **b**), the calculated specific lactate release rate (**c**) and the extracellular LDH activity (**d**) are given for the indicated time points (**a**, **b**, **d**) or incubation period (**c**). The data presented are means ± SD of values from experiments that had been performed on three independently prepared astrocyte cultures. The average protein content of the cultures was 154 ± 6 µg/well. In panel (**c**), the significance of differences (t-test) between data from incubations in the absence or the presence of 2DG for the given incubation periods (black vs. green and red vs. yellow) is indicated by ^#^p < 0.05, ^##^p < 0.01 and ^###^p < 0.001. Significant differences between data for the two different incubations periods of each condition (with or without 2DG preincubation, black vs. red and green vs. yellow) are indicated by ^+^p < 0.05 and ^++^p < 0.01

Control cells that had been preincubated for 30 min without 2DG efficiently produced and released lactate by glycolytic glucose metabolism in a time- and concentration-dependent manner (Fig. 3a). The extracellular lactate concentration accumulated for initial glucose concentrations of at least 0.5 mM almost proportional to time (Fig. 3a). Much less extracellular lactate was found for astrocytes that had been preincubated with 2DG for 30 min (Fig. 3b). The extracellular lactate accumulation in 2DG-preincubated cells was very slow for all glucose concentrations applied, at least for the initial 60 min of incubation (Fig. 3b) where the lactate accumulation rate accounted for around 35% of the values determined for the 2DG-free condition (Fig. 3c). However, during longer incubations the extracellular accumulation of lactate in 2DG-preincubated astrocytes was found substantially accelerated for all concentrations of glucose applied (Fig. 3c). None of the conditions applied compromised the viability of the cells as indicated by the very low activity of extracellular LDH for the conditions applied (Fig. 3d).

**Fig. 4:**
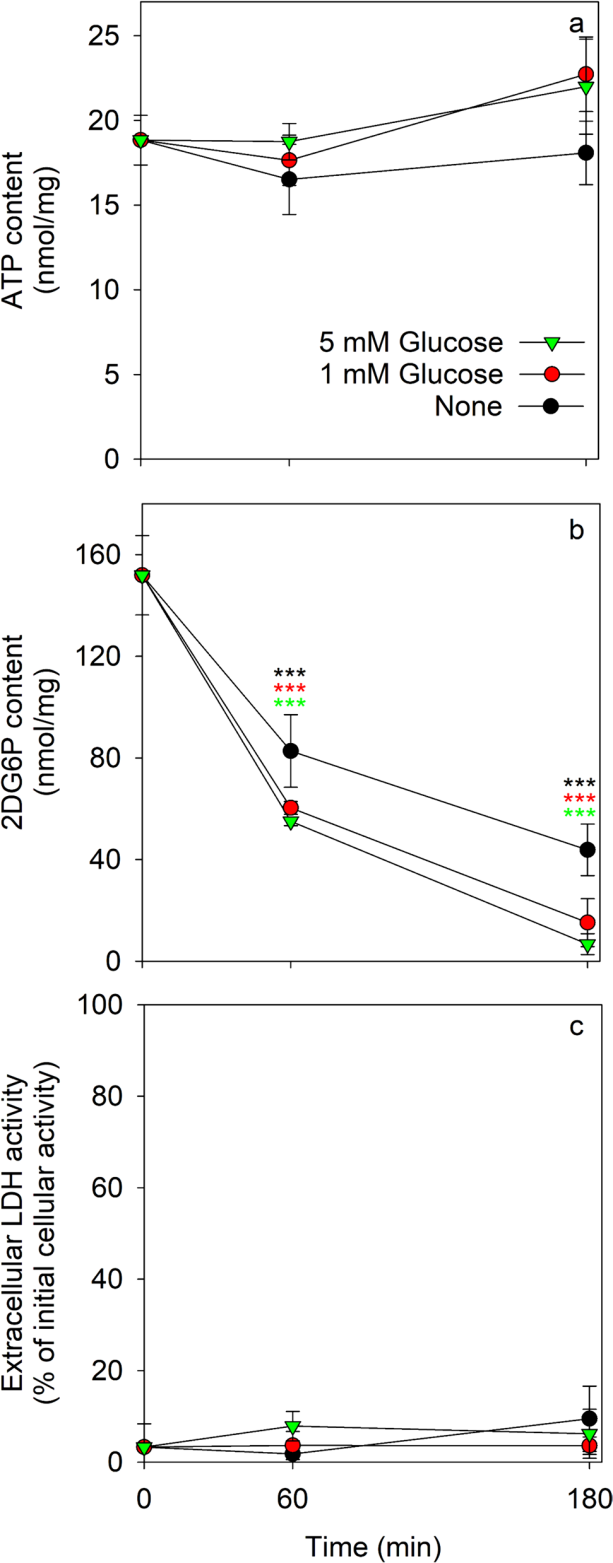
Cellular contents of ATP and 2DG6P during incubation of 2DG-preincubated astrocytes. The cells were pre-incubated for 30 min in 300 µL IB containing 10 mM 2DG, washed and subsequently incubated in 300 µL IB without (0 mM) or with 1 mM or 5 mM glucose. The specific cellular ATP (**a**) and 2DG6P (**b**) contents as well as the extracellular LDH activity (**c**; given as % of the initial cellular LDH activity) were determined for the indicated incubation periods. The data presented are means ± SD of values from experiments that had been performed on three independently prepared astrocyte cultures. The initial ATP and protein contents of the cultures were 36.3 ± 4.4 nmol/mg and 108 ± 11 µg/well, respectively. The significance of differences (ANOVA) compared to the data determined after the preincubation (0 min) is indicated by ***p < 0.001

The cellular ATP content of 2DG pre-incubated astrocytes was not significantly altered during a 2DG-free main incubation for up to 180 min in the absence or the presence of 1 mM or 5 mM glucose (Fig. 4a). In contrast, the initial high specific 2DG6P content of 2DG6P-loaded astrocytes was significantly lowered during the main incubations (Fig. 4b). After 60 min and 180 min main incubation in the absence of 2DG and glucose the cellular 2DG6P contents accounted for around 50% and 30% of the initial content, respectively, while the cellular 2DG6P content declined even quicker if glucose had been present during the main incubation of 2DG pre-loaded astrocytes (Fig. 4b). For these conditions, the cellular 2DG6P content accounted after 180 min main incubation to only 10% (1 mM glucose) and 4% (5 mM glucose) of the initial 2DG6P content (Fig. 4b). None of these conditions compromised the cell viability (Fig. 4c).

The cellular contents of 2DG and 2DG6P in the 2DG-preincubated astrocytes accounted for 151.9 ± 15.6 nmol/mg (2DG6P) and 53.1 ± 17.3 nmol/mg (free 2DG), which corresponds to a total cellular content of 205 nmol/mg 2DG plus 2DG6P (Table 3). During the main incubation in the absence of glucose the total amount of cellular 2DG plus 2DG6P was lowered by 50% and 70% within 60 min and 180 min, respectively (Table 3). Around 25% of the initial sum of 2DG plus 2DG6P were found extracellularly both after 60 min and 180 min of incubation, while at best marginal amounts of extracellular 2DG6P were found for these conditions (Table 3). In conclusion, glycolytic lactate production was initially impaired in 2DG6P-loaded astrocytes, but reestablished during longer incubation periods consistent with an observed loss in cellular 2DG6P content.

**Table 3:** Cellular and extracellular contents of 2DG and 2DG6P during the incubation of 2DG pre-treated astrocytes

Main incubation (min)	Cellular 2DG content (nmol/mg)	Cellular 2DG6P content (nmol/mg)	Extracellular 2DG content (nmol/mg)	Extracellular 2DG6P content (nmol/mg)	∑ (2DG + 2DG6P) contents (nmol/mg)
0	53.1 ± 17.3	151.9 ± 15.6	0	0	205
60	14.8 ± 3.1*	82.7 ± 14.3**	54.4 ± 13.9***	1.5 ± 1.7	153
180	18.9 ± 1.9*	43.8 ± 10.1***	57.6 ± 6.7***	0.8 ± 1.4	121

The cells were pre-incubated for 30 min with 300 µL IB containing 10 mM 2DG, washed and subsequently incubated in 300 µL IB without glucose and 2DG. The cellular and extracellular contents of 2DG and 2DG6P were determined after the indicated incubation periods. The data presented are means ± SD of values from experiments that had been performed on three independently prepared astrocyte cultures and were determined for the samples of the experiments presented in Fig. 4

The significance of differences (ANOVA) compared to the 0 min time point is indicated by the symbols *p < 0.05, **p < 0.01 and ***p < 0.001

### Consequences of a Pre-Loading of Astrocytes with 2DG6P on the NADPH- and PPP-Dependent WST1 Reduction

To test for the consequences of a high cellular concentration of 2DG6P on the NADPH regeneration by the PPP of astrocytes, the PPP- and NADPH-dependent WST1 reduction was investigated in the absence and the presence of glucose in the experimental paradigm previously described [26, 42]. After the 30 min preincubation of astrocytes in the presence of 2DG, the cells contained a specific 2DG6P content of 130.6 ± 9.1 nmol/mg. The initial specific cellular ATP content of 29.7 ± 5.4 nmol/mg had been lowered by 50% to 14.6 ± 2.9 nmol/mg during the preincu(Table 2). In contrast, after 30 min preincubation in the absence of 2DG the cellular ATP content of the cells was not significantly affected and as expected 2DG6P was not detectable in the cells (data not shown).

**Fig. 5:**
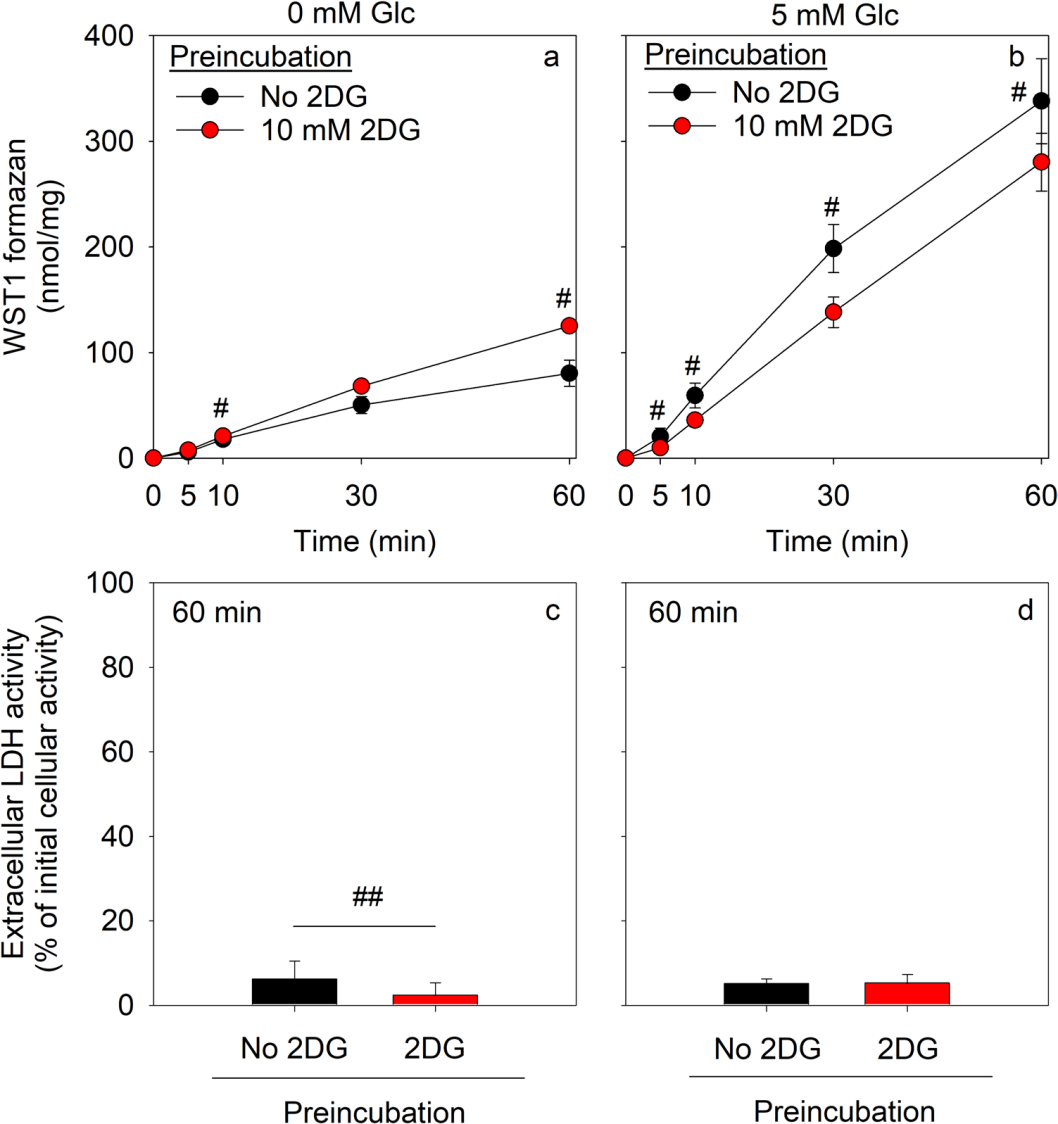
Effect of a preincubation with 2DG on the glucose-dependent β-lapachone-mediated WST1 reduction in cultured astrocytes. The cells were preincubated for 30 min in 400 µL glucose-free IB the absence (0 mM) or the presence of 10 mM 2DG, washed and subsequently incubated with 3 µM β-lapachone and 400 µM WST1 in the absence or the presence of 5 mM glucose. The extracellular WST1 formazan content (**a**, **b**) was measured after the indicated time points and the extracellular LDH activity (**c**, **d**) was determined after 60 min. The initial protein content was 155 ± 4 µg per well. The data presented are means ± SD of values from experiments that had been performed on three independently prepared astrocyte cultures. The significance of differences (t-test) between data from incubations in the absence or the presence of 2DG is indicated by ^#^p < 0.05 and ^##^p < 0.01

For astrocytes that had been pre-incubated in the absence of 2DG, almost linear extracellular accumulations of WST1 formazan were observed that reached within 60 min values of around 80 (Fig. 5a) and 340 (Fig. 5b) nmol/mg for incubations without and with glucose, respectively. For 2DG-preincubations, these values were found significantly increased by around 60% after 60 min of incubation in hexose-free IB (Fig. 5a), but lowered by around 30% for glucose-treated astrocytes (Fig. 5b). None of the conditions applied caused any substantial loss in cell viability as compared to the control incubations (Fig. 5c, d).

The lowered cellular ATP content of 2DG-treated astrocytes was not further modified by an incubation of astrocytes in the absence or the presence of glucose, β-lapachone and WST1 (Table 4), while the high initial cellular 2DG6P content declined during the 60 min main incubation without β-lapachone and WST1 by around 50% (absence of glucose) and 70% (presence of 5 mM glucose). A much stronger 2DG6P decline was found for incubations in the presence of β-lapachone plus WST1, as after 60 min of incubation the cellular 2DG6P contents accounted for only 5% of the initial content (Table 4). These data suggest that accumulated 2DG6P has only a rather low inhibitory potential for the PPP in glucose-fed astrocytes and that it may even serve as artificial substrate for G6PDH.

**Table 4:** Cellular contents of ATP and 2DG6P during incubation of astrocytes with β-lapachone and WST1 after a 2DG pre-incubation

Main incubation with [glucose] (mM)	Main incubation with β-lapachone + WST1	Incubation time (min)	Cellular ATP content (nmol/mg)	Cellular 2DG6P content (nmol/mg)	LDH release (%)
–	–	0	14.6 ± 2.5	130.7 ± 9.1	9 ± 11
0	No	60	13.0 ± 3.1	63.9 ± 3.7***	8 ± 10
0	Yes	60	11.8 ± 1.4	5.2 ± 9.0***^,###^	9 ± 12
5	No	60	13.6 ± 3.6	42.6 ± 7.1***	4 ± 8
5	Yes	60	14.1 ± 2.6	4.9 ± 4.4***^,##^	1 ± 3

The cells were pre-incubated for 30 min in 400 µL IB containing 10 mM 2DG, washed and subsequently incubated in 400 µL 2DG-free IB without or with 5 mM glucose in the absence or the presence of 3 µM β-lapachone plus 400 µM WST1. The specific cellular ATP and 2DG6P contents were determined before and after 60 min of incubation. The extracellular LDH activity (given as % of the initial cellular LDH activity) was determined after the 60 min main incubation. The data presented are means ± SD of values from experiments that had been performed on three independently prepared astrocyte cultures. The initial ATP and protein contents of the cultures were 29.8 ± 4.8 nmol/mg and 117 ± 14 µg/well, respectively

The significance of differences (ANOVA) compared to the respective 0 min time point is indicated by ***p < 0.001. The significance of differences (t-test) between data for respective incubations without and with β-lapachone plus WST1 during the main incubation are indicated by hashes with ^##^p < 0.01 and ^###^p < 0.001

### Test of Utilization of 2DG6P as Substrate for PPP-Dependent WST1 Reduction

**Fig. 6:**
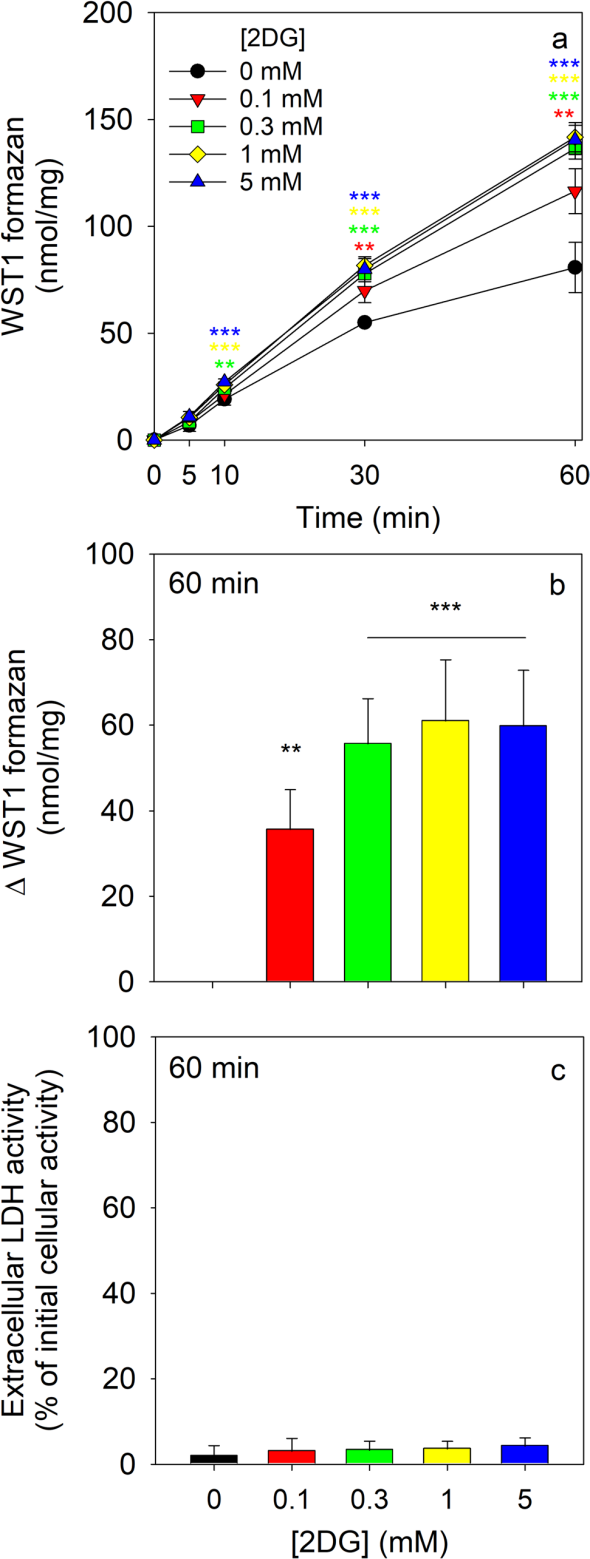
Use of 2DG as extracellular substrate for β-lapachone-mediated WST1 reduction in cultured astrocytes. The cells were preincubated in 400 µL hexose-free incubation buffer and subsequently incubated in 400 µL IB containing 3 µM β-lapachone, 400 µM WST1 and 2DG in the indicated concentrations. The extracellular WST1 formazan content was measured for the indicated time points (**a**), the 2DG-dependent WST1 reduction was calculated for the 60 min incubation period (**b**) and the extracellular LDH activity (**c**) was determined for the 60 min incubation period. For the control incubation in the presence of 5 mM glucose (not shown), an extracellular WST1 accumulation of 350 ± 18 nmol/mg was determined for the 60 min incubation. The initial protein content of the cultures was 154 ± 5 µg per well. The data presented are means ± SD of values from experiments that had been performed on three independently prepared astrocyte cultures. The significance of differences (ANOVA) compared with data for incubations without 2DG (0 mM) is indicated by **p < 0.01 or ***p < 0.001

To test for a potential utilization of 2DG6P as substrate for the NADPH- and PPP-dependent WST1 reduction, astrocytes were preincubated for 30 min in glucose-free buffer before 2DG was applied in different concentrations. An almost linear extracellular accumulation of WST1 formazan was observed for incubations in the absence of hexoses that had reached within 60 min values of around 80 nmol/mg (Fig. 6a). Application of 2DG caused a concentration-dependent increase in the detectable amount of extracellular WST1 formazan (Fig. 6a, b) that was almost doubled compared to that of the control incubation (absence of 2DG) for 2DG in concentrations of 0.3 mM or higher (Fig. 6a, b), suggesting that 2DG6P is a substrate for G6PDH and PPP in cultured astrocytes. None of the conditions applied caused any obvious toxicity as demonstrated by the absence of any increase in extracellular LDH activity (Fig. 6c).

### Consequences of a Coincubation of Astrocytes with Glucose and an Excess of 2DG on the NADPH- and PPP-Dependent WST1 Reduction

**Fig. 7:**
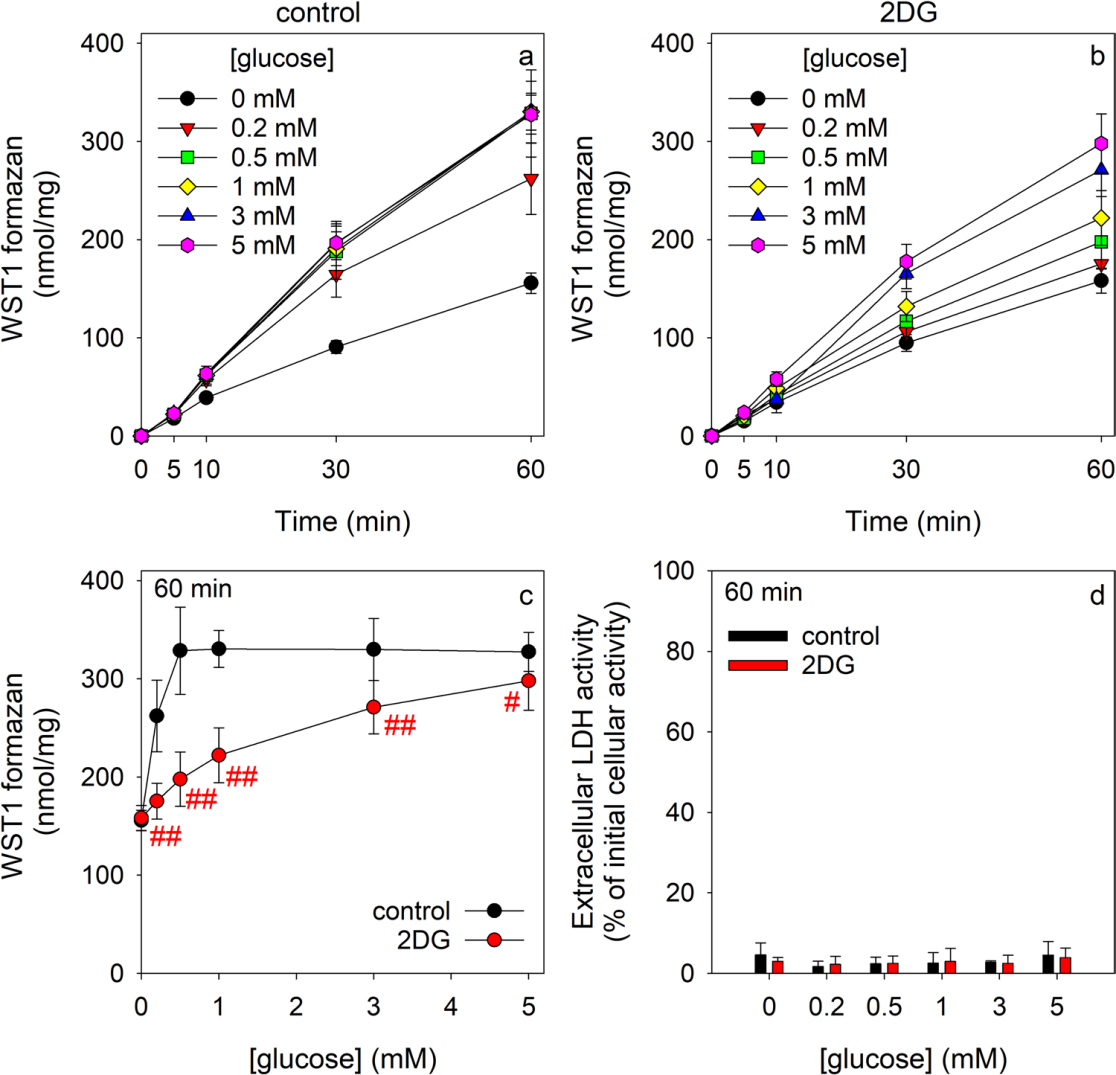
Modulation by an excess of 2DG of the glucose-dependent β-lapachone-mediated WST1 reduction by cultured astrocytes. The cells were incubated in 400 µL IB containing the given initial glucose concentrations in the absence (**a**, control) or the presence of 10 mM 2DG (**b**) with 3 µM β-lapachone and 400 µM WST1. The extracellular WST1 formazan content (**a**, **b**, **c**) was determined for the indicated time periods and the extracellular LDH activity (**d**) after 60 min of incubation. The data presented are means ± SD of values from experiments that had been performed on three independently prepared astrocyte cultures. The initial protein content of the cultures was 151 ± 15 µg/well. In panel (**c**), the significance of differences (t-test) between the data obtained for incubations in the absence and the presence of 2DG is indicated by ^#^p < 0.05 and ^##^p < 0.01

For astrocytes that had been incubated with glucose in the absence of 2DG, almost linear extracellular accumulations of WST1 formazan were observed that depended on the concentration of glucose applied, reached maximal values for an initial glucose concentration of 0.5 mM and accumulated within 60 min to values of around 120 and 320 nmol/mg for incubations without and with glucose, respectively (Fig. 7a). In contrast, the extracellular WST1 formazan accumulation of astrocytes that were coincubated with glucose in the presence of an excess of 2DG was slower (Fig. 7b) compared to data for the 2DG-free incubations (Fig. 7a) and after 60 min significantly less WST1 formazan was determined for 2DG-exposed astrocytes for each initial glucose concentration applied (Fig. 7c). This demonstrates that the presence of an excess of 2DG slows the utilization of extracellular glucose as substrate for the PPP. None of the conditions applied caused any substantial loss in cell viability as compared to the control incubations (Fig. 7d).

### Consequences of a Coincubation of Astrocytes with Glucose and an Excess of 2DG on the Glycolytic Lactate Production and the Cellular Contents of ATP and 2DG6P

**Fig. 8:**
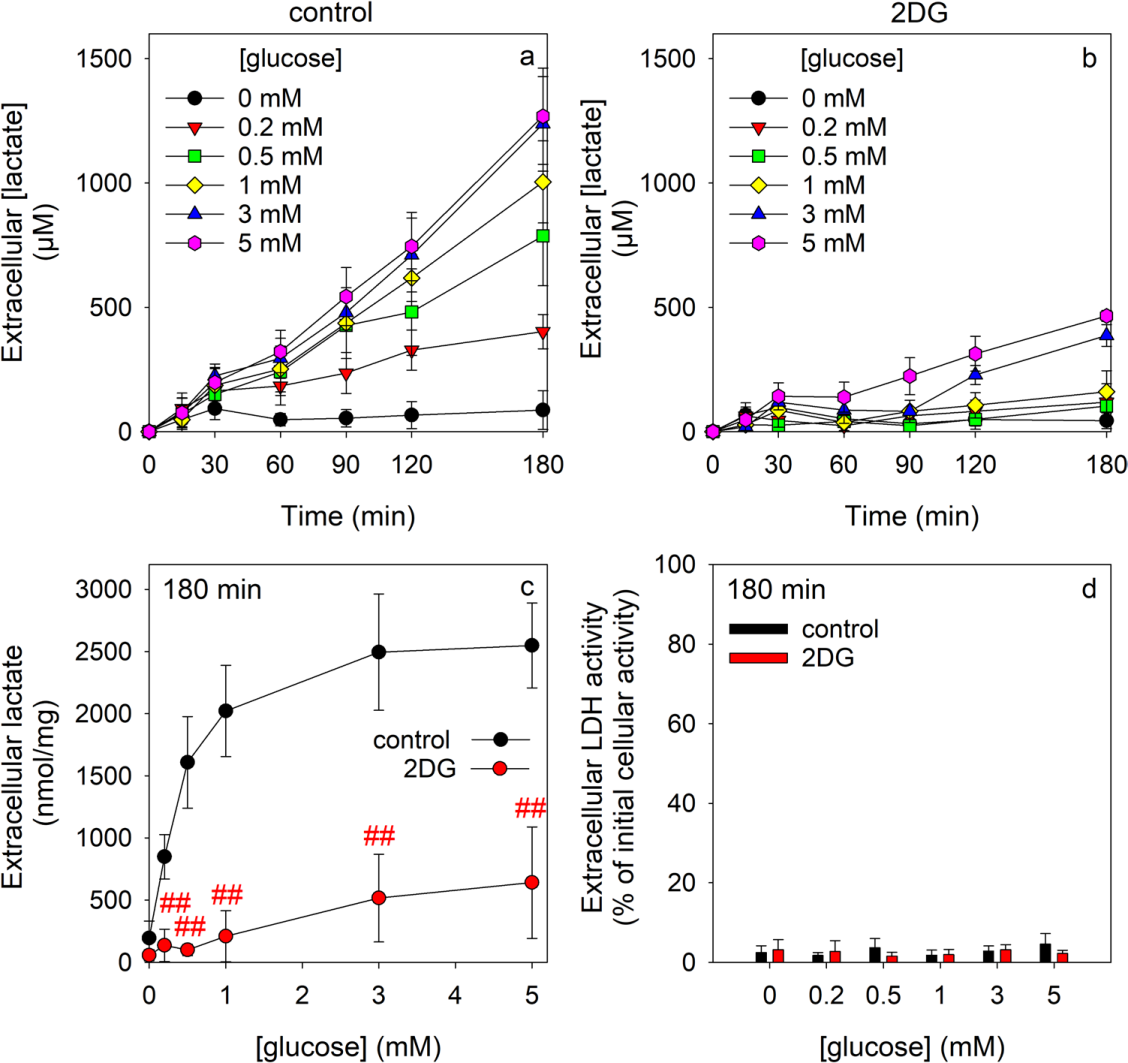
Modulation by an excess of 2DG of the glucose-dependent lactate release from cultured astrocytes. The cells were incubated for up to 180 min in 300 µL IB containing glucose in the given initial concentrations in the absence (**a**, control) or the presence of 10 mM 2DG (**b**). The extracellular lactate concentration (**a**, **b**) was determined for the indicated time periods and the specific extracellular lactate content (**c**) as well as the extracellular LDH activity (**d**) were determined after 180 min of incubation. The data presented are means ± SD of values from experiments that had been performed on three independently prepared astrocyte cultures. The initial protein content of the cultures was 132 ± 13 µg/well. In panel (**c**), the significance of differences (t-test) between the data obtained for incubations in the absence and the presence of 2DG is indicated by ^##^p < 0.01

**Fig. 9:**
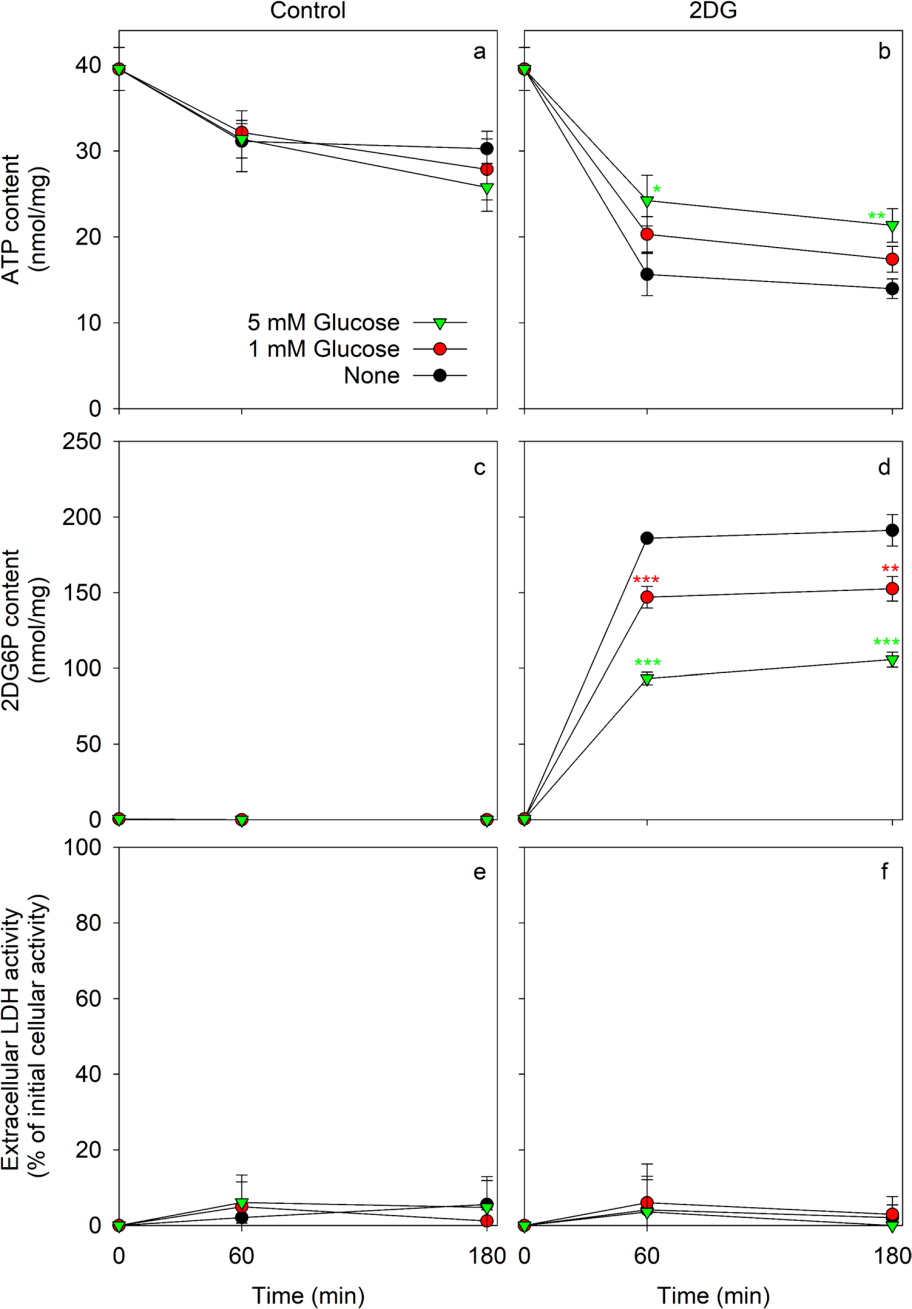
Cellular contents of ATP and 2DG6P during coincubations of astrocytes with glucose and an excess of 2DG. The cells were incubated for up to 180 min in 300 µL IB containing 0 mM or 10 mM 2DG in the absence or the presence of 1 mM or 5 mM glucose. The specific cellular ATP (**a**, **b**) and 2DG6P (**c**, **d**) contents as well as the extracellular LDH activity (**e**, **f**) (given as % of the initial cellular LDH activity) were determined after the indicated incubation periods. The data presented are means ± SD of values from experiments that had been performed on three independently prepared astrocyte cultures. The initial ATP and protein contents of the cultures were 39.5 ± 2.5 nmol/mg and 94 ± 23 µg/well, respectively. The significance of differences (ANOVA) compared to the respective incubations without glucose is indicated by the symbols *p < .0.05, **p < 0.01 and ***p < 0.001

Glucose-exposed astrocyte cultures that were incubated without 2DG efficiently produced and released lactate by glycolytic glucose metabolism in a time- and concentration-dependent manner that was almost proportional to time (Fig. 8a). Much less extracellular lactate was found for astrocytes that had been incubated with glucose in concentrations between 0.5 and 5 mM in the presence of 10 mM 2DG (Fig. 8b). The specific lactate accumulation followed for glucose-fed astrocytes that had been incubated in the absence of 2DG a glucose concentration-dependent hyperbolic function. In contrast, the extracellular lactate accumulation determined after 180 min incubations in the presence of 10 mM 2DG increased almost linearly with the glucose concentration applied, but accounted even for incubations with 5 mM glucose to only 25% of the amount found for the respective 2DG-free condition (Fig. 8c). None of the conditions applied compromised the viability of the cells as indicated by the very low activity of extracellular LDH for the conditions applied (Figs. 8d, 9e, f).

The cellular ATP content of astrocytes that had been incubated without glucose was not severely affected during an incubation for up to 180 min with 1 mM or 5 mM glucose (Fig. 9a). In contrast, for coincubations of astrocytes without or with 1 mM or 5 mM glucose in the presence of 10 mM 2DG, the ATP contents were lowered within 60 min to around 40%, 50% and 60% of the initial ATP content, respectively (Fig. 9b). For these conditions, cellular 2DG6P had accumulated within 60 min to values of 186 ± 1 nmol/mg (0 mM glucose), 147 ± 7 nmol/mg (1 mM glucose) and 93 ± 4 nmol/mg (5 mM glucose). Longer incubations for 180 min hardly affected the cellular contents of ATP or 2DG6P compared to the values found for a 60 min incubation (Fig. 9b, d). These data demonstrate that strong 2DG6P accumulation and ATP loss are also found after exposure of glucose-fed astrocytes to 2DG.

## Discussion

Application of 2DG to cultured astrocytes in glucose-free buffer caused a rapid initial decline in cellular ATP content which is consistent with literature data [19, 20]. The known rapid phosphorylation of 2DG to 2DG6P in astrocytes [13, 14] is likely to substantially contribute to the observed initial decline of cellular ATP in 2DG-treated astrocytes. The 2DG-induced ATP loss accounted for around 50% within 30 min of incubation, but during longer incubations with 2DG the cellular ATP content remained rather stable and only a slow further decline was observed to around 40% and 30% of the initial value after 3 h and 24 h of incubation, respectively. None of the conditions applied in the current study caused any obvious cell toxicity despite the observed severe loss in cellular ATP content. However, cellular ATP contents in 2DG-treated cells were even after 24 h of incubation above the critical threshold level of ATP (around 25% of the normal ATP content) that is needed to prevent toxicity of cultured astrocytes [18, 43].

Despite the initial consumption of ATP for 2DG phosphorylation, astrocytes will continuously consume ATP for various additional cellular processes [18, 20, 31, 44]. Therefore, efficient and continuous ATP regeneration is required in 2DG-treated astrocytes. As glycogen mobilization [15] and glycolytic lactate production are inhibited in 2DG-treated astrocytes, ATP regeneration has to mainly rely on mitochondrial respiration. Indeed, inhibition of mitochondrial ATP regeneration by inhibitors of the respiratory chain (rotenone, antimycin A) or of the ATP synthase (oligomycin) or the presence of the mitochondrial uncoupler BAM15, lowered rapidly the cellular ATP content in glucose-deprived astrocytes. For all these conditions a co-application of 2DG strongly accelerated the cellular loss of ATP as previously shown for astrocytes treated with combinations of 2DG with rotenone [19] or antimycin A [20], supporting the view that mitochondrial ATP regeneration is the pathway that enables 2DG-treated astrocytes to maintain a cellular ATP concentration that is sufficiently high to keep the cells alive.

Both the cellular ATP and cellular 2DG6P levels remained almost constant after at least 60 min of incubation, but almost an order of magnitude more 2DG6P had accumulated in the 2DG-treated cells compared to the observed ATP loss, demonstrating that substantial ATP regeneration takes place in 2DG-treated astrocytes. As mentioned above, mitochondrial respiration is likely to fuel this process, since also the co-application of inhibitors of the uptake of mitochondrial pyruvate transporter and the carnitine shuttle for the uptake of activated fatty acids into mitochondria compromised the maintenance of a partial ATP content in 2DG-treated astrocytes as previously shown for glucose-deprived astrocytes [18].

After an initial rapid accumulation in the minute time frame, the level of cellular 2DG6P in 2DG-treated cells remained almost constant after 60 min of exposure, suggesting a steady state between cellular uptake and phosphorylation of 2DG by hexokinase, which will be inhibited by accumulating 2DG6P [45, 46], and consumption of accumulated 2DG6P by hydrolases and/or oxidases. The high cytosolic steady state concentrations of 20–30 mM and 30–40 mM 2DG6P determined for astrocytes that had been treated with 2DG in the presence and the absence of glucose, respectively, demonstrate that formation of 2DG6P is substantially quicker than cellular consumption of the accumulated 2DG6P. The lower accumulation of 2DG6P and the disappearance of ATP in 2DG-treated astrocytes that were incubated in the presence of glucose was expected due to the competition of 2DG with glucose for uptake and cellular phosphorylation. The accumulated high 2DG6P concentration in 2DG exposed astrocytes is likely to prevent further phosphorylation of 2DG by hexokinase as 2DG6P is known as competitive inhibitor of mammalian hexokinase [45, 46]. While for cultured astrocytes the uptake of 2DG [11, 12] and its phosphorylation [13, 14] are well known, less information is available on the fate of the accumulated 2DG6P in astrocytes. Most of the cellular 2DG6P that had been generated within a 30 min loading period in the presence of 2DG had disappeared from the cells within 3 h after removal of exogenous 2DG, confirming that accumulated 2DG6P is indeed metabolized in astrocytes. The disappearance of 2DG6P may in part be mediated by hydrolysis to free 2DG as previously reported for cultured astrocytes [13] but only a part of the metabolized cellular 2DG6P was found extracellularly as free 2DG. The loss of cellular 2DG6P was even accelerated during incubation with glucose, most likely due to competition by glucose for re-uptake of released 2DG and subsequent phosphorylation by hexokinase.

An additional pathway that may contribute to the disappearance of 2DG6P from cultured astrocytes is the oxidation of 2DG6P by G6PDH, as 6-phospho-2-deoxygluconate has been found in the brain of 2DG-exposed rats [4]. We have previously shown that cellular NADPH regeneration via the PPP can be studied by monitoring the NQO1-dependent reduction of extracellular WST1 to its formazan in the presence of the redox cycler β-lapachone [26, 42]. In the presence of 2DG the glucose-independent WST1 reduction was almost doubled in a concentration-dependent manner by 2DG, suggesting that 2DG6P acts as substrate of G6PDH in living astrocytes, thereby providing electrons via NADPH for the WST1 reduction. Also, the accelerated disappearance of 2DG6P from 2DG-preincubated astrocytes for conditions of intensified NADPH regeneration by G6PDH for β-lapachone-mediated WST1 reduction (compared to the β-lapachone-free condition), supports the view that, under the conditions used, cellular 2DG6P is oxidized by G6PDH. However, 2DG6P appears to be a rather poor substrate of G6PDH as even at cellular concentrations of 30 mM only around 20% of the NADPH regeneration was found compared to values determined for a glucose treatment. These data are consistent with literature reporting the rather slow oxidation of 2DG6P as substrate of murine G6PDH [47]. In addition, accumulated 2DG6P appears to have only a low inhibitory potential on the glucose-dependent NADPH regeneration as demonstrated by the observations that the glucose-dependent WST1 reduction in 2DG6P-preloaded astrocytes was only lowered by around 20% and that in coincubation experiments even a 20-fold excess of 2DG (10 mM) compared to glucose (0.5 mM) lowered cellular WST1 reduction only by around 40%.

The WST1 reduction method that we have used to investigate the potential of accumulated 2DG6P to serve as substrate for the PPP or to inhibit the use of G6P by G6PDH is based on the NADPH-dependent reduction by NQO1 of the electron cycler β-lapachone that subsequently permeates in reduced form the cell membrane and reduces extracellular WST1 [26, 42, 48]. This indirect method allows conclusions on the electron flow through the PPP but gives no information on the cellular level of NADPH. Further studies are now required to investigate whether a 2DG treatment of astrocytes may directly affect cellular levels of NADPH or NADP^+^ or their ratio, for example by applying quantitative methods to determine the cellular contents of NADP(H) [49] or by visualizing cellular NADPH contents by appropriate genetically encoded sensors [50].

In summary, a treatment of cultured astrocytes with 2DG and the rapid cellular accumulation of 2DG6P severely impairs glycolytic breakdown of glucose but has less severe effects on the NADPH regeneration from glucose via the G6PDH and does not compromise cell viability. The rapid loss of cellular ATP, which is connected to the initial phosphorylation of uptaken 2DG, lowers cellular ATP levels only partially due to the efficient mitochondrial ATP regeneration. Furthermore, the strong accumulation of cellular 2DG6P and its inhibitory potential on glycolysis can be reversed as cellular 2DG6P concentrations rapidly decline after removal of extracellular 2DG.

2DG has been described as glycolysis inhibitor [22, 51], but this appears not to be appropriate. In fact, 2DG competes with glucose for cellular uptake and phosphorylation, but it is not 2DG which affects glycolysis but rather the accumulated 2DG6P that inhibits hexokinase and hexosephosphate isomerase [45, 46, 52]. For glucose-fed cultured astrocytes the presence of an excess of 2DG lowered severely lactate production demonstrating that glycolysis was strongly affected by the high cellular concentrations of accumulate 2DG6P. However, it should be considered that a 2DG treatment does not specifically affect glycolysis. At least for cultured astrocytes an exposure to 2DG also lowers the cellular contents of ATP, creatine phosphate and the sum of adenosine phosphates [20], prevents glycogen mobilization due to an inhibition of glycogen phosphorylase by accumulated 2DG6P [53] and lowered at least to some extent glucose-6-phosphate oxidation by the PPP. Nevertheless, the 2DG-induced ATP loss and the other consequences of a 2DG treatment were not toxic for cultured astrocytes under the conditions investigated. In addition, the accumulated cellular 2DG6P level depended on the applied concentrations of 2DG and of glucose and the high cellular content of 2DG6P disappeared after removal of extracellular 2DG. These observations suggest that a transient 2DG treatment and a transient cellular accumulation of 2DG6P do not severely harm cells such as astrocytes.

Most of the data provided in this study are derived from experiments that have exposed cultured astrocytes to 2DG in the absence of glucose. However, a substantial loss in cellular ATP and a strong accumulation of 2DG6P was also observed for incubations of cultured astrocytes with glucose and 2DG, condition which are more likely to occur in the brain during 2DG-treatment. Thus, the observed alterations of cellular metabolism in cultured brain cells after 2DG exposure as well as the ability of 2DG to affect in addition to glycolysis also other metabolic processes should be considered in the context of a potential therapeutic use of 2DG as an anticancer drug [1, 21] as a drug for treatment of status epilepticus [22, 23] or as a treatment to limit inflammation in brain [17].

## Data Availability

Enquiries about data should be directed to the corresponding author.
